# Role of the C-terminal domain in the structure and function of tetrameric sodium channels

**DOI:** 10.1038/ncomms3465

**Published:** 2013-09-19

**Authors:** Claire Bagnéris, Paul G. DeCaen, Benjamin A. Hall, Claire E. Naylor, David E. Clapham, Christopher W. M. Kay, B. A. Wallace

**Affiliations:** 1 School of Biological Sciences, Institute of Structural and Molecular Biology, Birkbeck College, University of London, London WC1E 7HX, UK; 2 Howard Hughes Medical Institute, Department of Cardiology, Children’s Hospital Boston, Boston, Massachusetts 02115, USA; 3 Department of Neurobiology, Harvard Medical School, Boston, Massachusetts 02115, USA; 4 Microsoft Research Cambridge, Cambridge CB1 2FB, UK; 5 Institute of Structural and Molecular Biology, University College London, London WC1E 6BT, UK; 6 London Centre for Nanotechnology, University College London, London WC1H 0AH, UK; 7 These authors contributed equally to this work

## Abstract

Voltage-gated sodium channels have essential roles in electrical signalling. Prokaryotic sodium channels are tetramers consisting of transmembrane (TM) voltage-sensing and pore domains, and a cytoplasmic carboxy-terminal domain. Previous crystal structures of bacterial sodium channels revealed the nature of their TM domains but not their C-terminal domains (CTDs). Here, using electron paramagnetic resonance (EPR) spectroscopy combined with molecular dynamics, we show that the CTD of the NavMs channel from *Magnetococcus marinus* includes a flexible region linking the TM domains to a four-helix coiled-coil bundle. A 2.9 Å resolution crystal structure of the NavMs pore indicates the position of the CTD, which is consistent with the EPR-derived structure. Functional analyses demonstrate that the coiled-coil domain couples inactivation with channel opening, and is enabled by negatively charged residues in the linker region. A mechanism for gating is proposed based on the structure, whereby splaying of the bottom of the pore is possible without requiring unravelling of the coiled-coil.

## Abstract (web summary)

Prokaryotic voltage-gated sodium channels possess dynamically disordered cytoplasmic C-terminal domains. Bagnéris *et al*. present the structure of the *Magnetococcus marinus* NavMs pore and C-terminal domain and reveal its role in coupling channel inactivation and opening.

## Graphical abstract

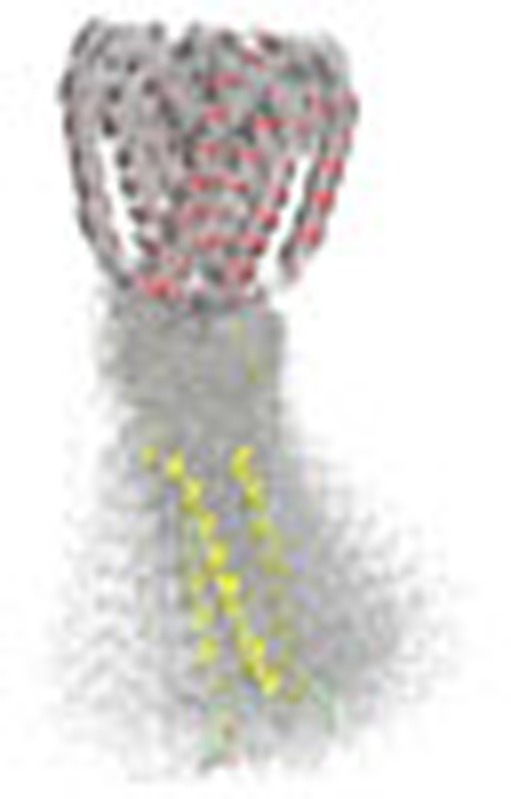

Voltage-gated sodium channels (VGSCs) are responsible for transmembrane (TM) sodium ion conduction and, in eukaryotes, for electrical signalling in excitable cells^1^. In humans, mutations in different sodium channel isoforms have been shown to have causal relationships with neurological and cardiovascular diseases, and they are therefore key targets for the development of pharmaceutical drugs. Eukaryotic sodium channels are comprised of single polypeptide chains with four homologous repeats that form pseudotetrameric channels^2^.

VGSCs are also present in a wide range of prokaryotes, where they have roles in motility and chemotaxis^3^. They are tetramers^4^, composed of identical monomers, which exhibit sequence homology to the individual eukaryotic repeats. More than 30 prokaryotic orthologues have been identified from bacterial sources, mostly from extremophiles. In each, the monomers consist of a TM domain that contains the voltage sensor and pore functionalities, and a C-terminal domain (CTD) that may have a role in assembly of the tetrameric channels^5^,^6^.

Crystal structures have been reported for several prokaryotic sodium channels. The different structures represent different functional states; they include both a mixed closed pore/open voltage sensor form^7^ and a potentially inactivated form of the NavAb orthologue^8^, an open pore form of the closely related NavMs orthologue^9^ and a proposed inactivated form of the more distantly related NavRh orthologue^10^. All of the constructs used to produce these structures included at least part of the CTDs, but in none of them was any of the CTD visible (i.e. not beyond the end of the last TM helix (designated S6)). Using synchrotron radiation circular dichroism spectroscopy, Powl *et al*.^5^ showed that the secondary structure of the CTD of the NaChBac orthologue (Fig. 1a) consists of a proximal unordered region, followed by a distal helical region. Sequence analyses^5^,^6^ suggested that the distal helical region would form a coiled-coil, based on the presence of the characteristic heptad repeat; the same sequence motif has been found in all prokaryotic sodium channel sequences examined to date, as well as in many related eukaryotic ion channels such as CatSper^11^, TRPV1 (ref. 12) and Kv7 (ref. 13). More recently, the crystal structure of a chimeric construct consisting of part of the distal end of the CTD of another NaChBac orthologue, NavSulP, attached to the end of an existing coiled-coil region of an unrelated NaK channel^14^ (Fig. 1a), showed it could form a coiled-coil in that context, but it was missing the important proximal linker region between the coiled-coil and TM domain. Hence, there is as yet no experimental evidence for either the three-dimensional structure or function of such a CTD in an intact sodium channel or any other related tetrameric prokaryotic or eukaryotic ion channel.

In this study, we examine the CTD as part of the NavMs bacterial sodium channel pore^9^, using continuous wave electron paramagnetic resonance (cw-EPR) and pulsed double electron–electron resonance (DEER) spectroscopy to define the supersecondary/quaternary structure of the domain. The distance distributions obtained for labelled sites in the CTD, combined with coarse grained molecular dynamics simulations, enable a model of this region to be constructed. These studies show that the end of the TM pore is connected to a four-helix coiled-coil bundle through a short linker region that, although flexible, is not simply disordered. This EPR-derived structure is then compared with a new crystal structure of the NavMs pore containing a full-length CTD; the location, but not the detailed structure, of the CTD could be deduced from the crystal electron density map, which is compatible with the EPR-derived structure. Electrophysiology measurements show that the CTD is necessary for inactivation gating and recovery, and for channel stability. To account for the functional effects, we propose that the linker and coiled-coil C-terminal region resemble an oscillator composed of a spring tethered to a mass that enables opening of the gate without uncoiling the coiled-coil or dissociating the tetramers.

## Results

### Sequence features

**Figure 1:**
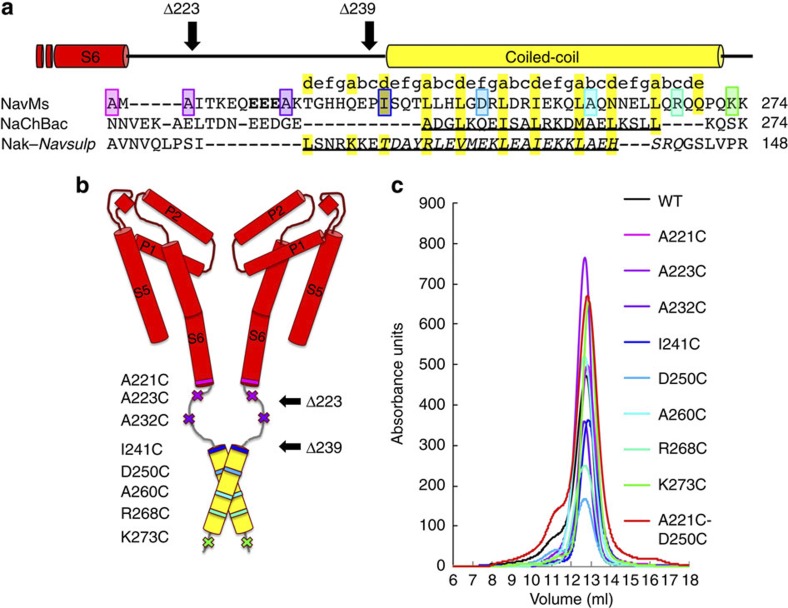
Labelled proteins. (**a**) Alignment of the CTDs of bacterial sodium channels starting at the end of transmembrane helix S6 (red bar above alignment). GenBank nucleotide accession codes: NavMs, YP_864725; NaChBac, NP_242367; chimeric NaK-NavSulP, with NaK, AB617622 and (italicised) NavSulP, NAS-14.1, AALZ01000002. The coiled-coil region predicted for NavMs is indicated by a yellow bar above the sequence alignment. The residues in NaChBac, which form the helical region^5^ and the region of the NaK-NavSulP chimera, which forms a coiled-coil^14^ are underlined. Residues that were mutated to cysteines and spin labelled are in coloured boxes. The same colouring scheme for each mutant is used in all figures: transmembrane residue A221 is pink, linker region residues A223 and A232 are lilac and purple, respectively; residues I241, D250, A260 and R268 in the predicted coiled-coil region are shades of blue and the final residue K273 is green. The EEE residues changed in the QQQ mutant used for electrophysiology are in bold. (**b**) Model of the open pore channel, adapted from McCusker *et al.*^9^ showing the locations of the spin-labelled amino acids. The outward displacement of the end of transmembrane helix S6 in this open form is due to a kink at T209 in the middle of S6. Only two of the four monomers are shown for clarity. The black arrows indicate the positions of the truncations (Δ223 and Δ239) used in the electrophysiology experiments. (**c**) Size exclusion chromatography of wild-type and mutant proteins used in the EPR experiments, showing all run as well-behaved tetramers.

The NavMs CTD possesses a typical heptad repeat of (a-b-c-d-e-f-g) between residues 240 and 270, where a and d are hydrophobic residues and e and f are hydrophilic residues (Fig. 1a), suggesting this region forms a coiled-coil. The linker region between residues 222 and 239 is predicted to have no regular secondary structure, consistent with the experimentally determined^5^ secondary structure of the NaChBac orthologue (Fig. 1a).

### Structure and dynamics by EPR spectroscopy and simulations

**Figure 2:**
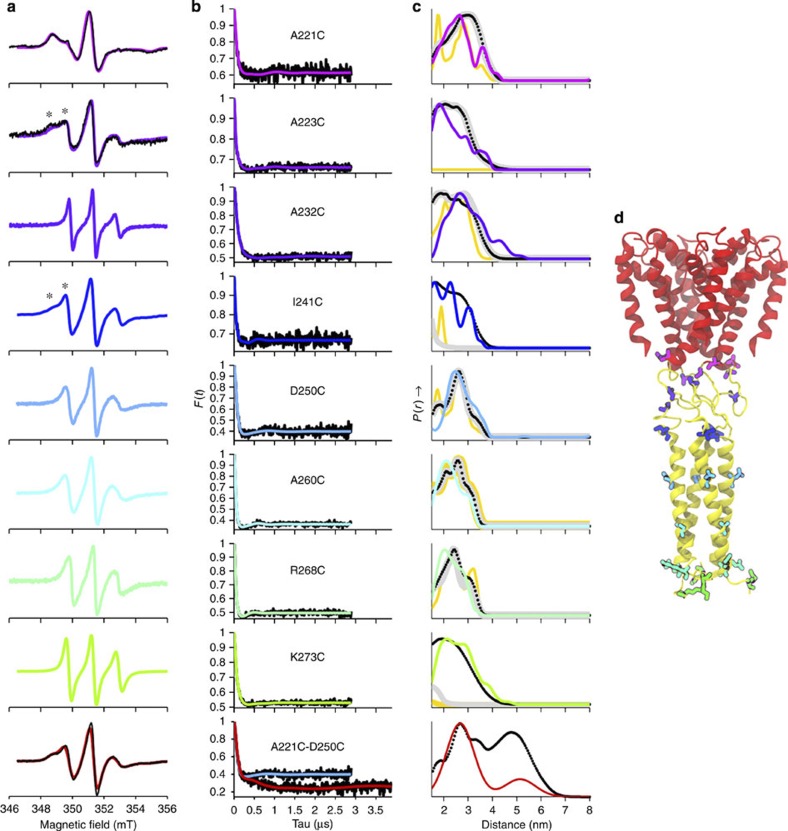
EPR spectroscopy. (**a**) The first derivative absorption cw-EPR spectra of PROXYL spin-labelled detergent-solubilized NavMs constructs are depicted in coloured lines, with A221C and A223C spin-labelled mutants in a liposome environment overlaid (black lines) in the top two panels. Additional shoulders present in the low-field region for A223C and I241C are indicated by ‘*’. In the bottom panel, the spectrum of the A221C/D250C double mutant (red line) is compared to the 1:1 summation (black line) of the spectra of the A221C and D250C single mutants. Spectra were normalized to reflect approximately equal numbers of spins. (**b**) Background-corrected dipolar evolution data (black lines) and the fits to the DEER data obtained by Tikhonov regularization (coloured lines). (**c**) Distance distributions obtained by: Tikhonov regularization (different coloured lines for each mutant spectrum), except for the A221C–D250C double mutant (bottom panel) where two Rice distributions were used; MMM predictions based on the first static model structure are represented as orange lines and those based on the first dynamic model are thick grey lines; MMM predictions based on the final dynamic model are in black dotted lines. All plots are normalized by amplitude. (**d**) Three-dimensional structure of the NavMs transmembrane pore crystal structure with the CTD as determined by the DEER spectroscopy/molecular dynamics approach.

Amino acids in the NavMs pore with its full-length CTD (NavMs-pore+CTD) construct, starting from the end of helix S6, through the putative unordered linker region, and along the predicted coiled-coil region, were mutated to cysteines to enable attachment of the nitroxide spin label (3-(2-iodoacetamido)-PROXYL) (Fig. 1a,b and Supplementary Fig. S1). All labelled proteins, as assessed by size exclusion chromatography (Fig. 1c), had similar tetrameric elution profiles to that of the unlabelled wild-type construct.

cw-EPR spectroscopy (Fig. 2a) provided information about the mobility of the spin labels and thus about the local environments of the labelled residues^15^. The label on A221C, located at the end of helix S6, appears to be highly immobilized, as would be expected for a label on a crowded, rigid part of a protein. The spectrum for A232C (in the middle of the linker region) has narrow lines, indicating the label is located on a highly mobile part of the protein. In contrast, the A223C and I241C labels (at opposite ends of the linker region) have two shoulders, rather than one, on the low-field region (indicated by ‘*’ in Fig. 2a), suggesting either that they possess two distinct rotamer conformations, or more likely, they experience two distinct environments: one more restricted (hence slower motion) and one less restricted (hence faster motion). A likely explanation is that these labels spend some time in a helix and some time in another more flexible structure. The labels at D250C, A260C and R268C (in the predicted four-helix bundle) display relatively restricted mobilities. Finally, the labels at K273C have a somewhat more mobile but also slightly broadened spectrum, which is reasonable, as the labels are adjacent to the final CTD residue and therefore would experience more flexibility, potentially bringing them into close proximity with each other.

Four-pulse DEER experiments enabled measurements of distance distributions between pairs of spin labels based on dipolar interactions^16^. As the NavMs-pore+CTD is composed of four identical spin-labelled monomers in a tetrameric structure, each spin experiences more than one interaction. In the simplest case, a symmetric tetramer, the labels would form a square with distances arising from the four sides and the diagonals of the square, the latter being √2 times longer than the sides (but their signals should be half the intensity). The background-corrected dipolar evolutions (black lines) from the eight spin-labelled mutants and their derived fits (coloured lines), known as form factors (Fig. 2b, with raw data in Supplementary Fig. S2), give rise to corresponding distance distributions (coloured lines) (Fig. 2c). The labels at A221C, D250C, A260C and R268C all show a single major distance peak and a lower intensity shoulder (roughly half the amplitude of the former) at √2 times the distance. These results suggest the labels experience a highly symmetrical arrangement consistent with a rigid tetrameric structure at the base of the pore and along the central part of the proposed four-helix bundle. Labels A223C, A232C, I241C and K273C, show broader distributions with three major peaks arising from the presence of a larger range of conformations. Consistent with the cw-EPR data, this indicates a decreased symmetry and less well-defined structure in the linker region and at the C-terminus of the CTD.

**Figure 3:**
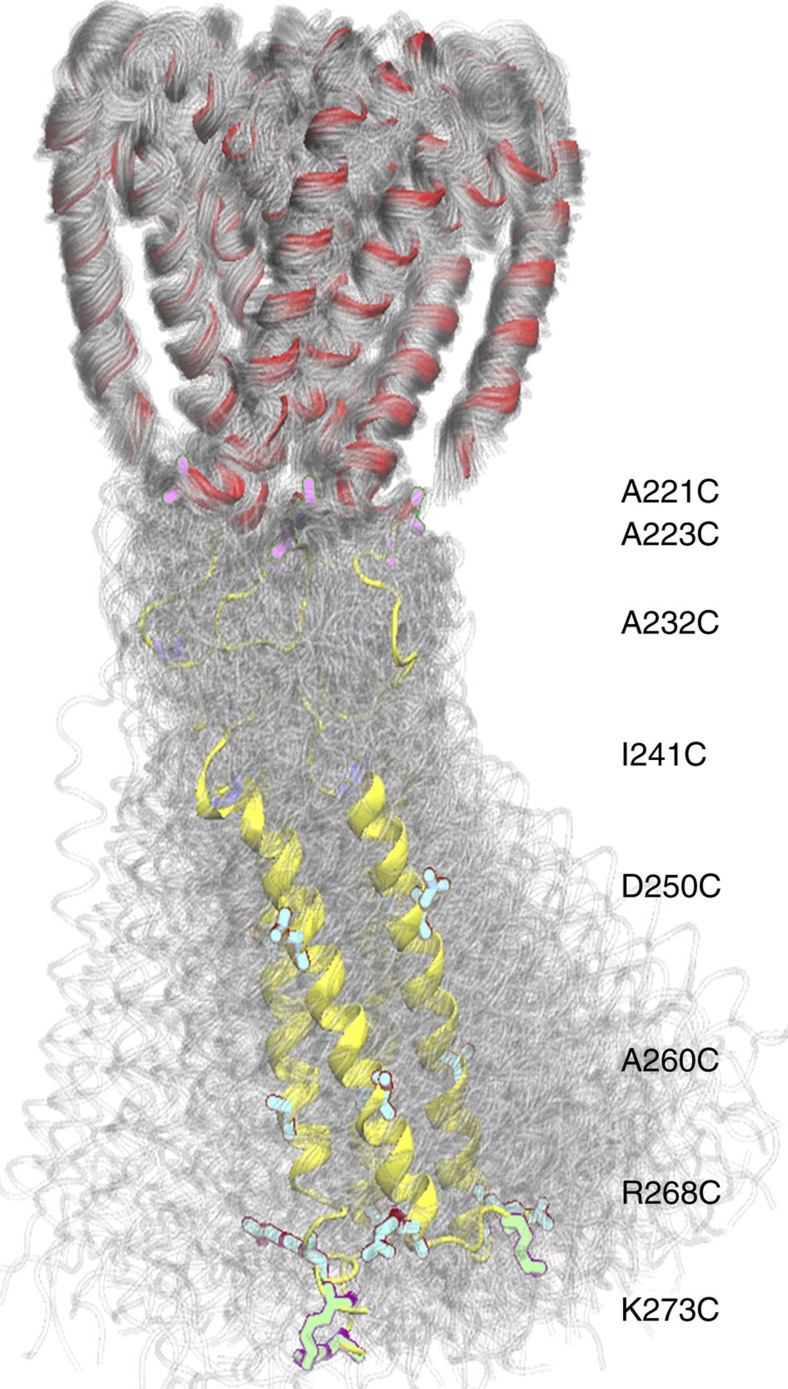
DEER-derived model showing the dynamics of the pore and CTD. Individual structures from separate trajectories are overlaid to show the large motions in the CTD, clearly indicating why this region is not well ordered in the crystal electron density map. See also Supplementary Movie 1.

Comparison of the distance distributions between spin labels derived from DEER experiments were made with predictions using the MMM approach^17^. The rotamer library and populations used by MMM are derived from detailed, extended molecular dynamics simulations and have been shown to be capable of high-resolution discrimination between distance distributions; as such, they can provide a straightforward means of verifying *in silico* models. Initial comparisons of the distance distributions of the spin labels derived from the DEER experiments in this study (Fig. 2c, differently coloured lines for each site) with MMM predictions^17^ (Fig. 2c, orange lines) using a static model of a fully coiled-coil four-helix bundle structure (Supplementary Figs S3 and S4a), suggested that the agreement was good for A221C at the base of the pore and for D250C, A260C and R268C in the coiled-coil, confirming the expected square symmetry. However, for labels that were mobile (A223C, A232C and I241C in the flexible region and K273 at the C-terminus) the agreement was, unsurprisingly, relatively poor. To explicitly take into account of the flexibility of the protein, a coarse-grained simulation of the model was performed in a dipalmitoylphosphatidylcholine bilayer, followed by conversion to an all-atom representation. Residues A221 to I240 in the dynamic model were first modelled as helical, but allowed to relax to unstructured coils (Supplementary Fig. S4b). The resulting model distance distributions for A223C (Fig. 2c, thick grey lines) reasonably estimated the broad distance distribution observed, however, for A221C at the base of the pore, the ensemble of distributions clearly overestimated geometries that the spin-label could sample, as the bimodal distribution is lost. Also, for I241C on the other side of the flexible region and for K273C at the C-terminus, the prediction was still not acceptable. Therefore, the latter residues at both ends of the coiled-coil region were also allowed to relax in the final model. The resulting distributions (Fig. 2c dotted black lines) for I241C and K273C were improved, producing an acceptable predicted distance distributions for all sites, and showing that the region between A223 and I241 is highly flexible, but not disordered. An impression of the dynamic nature of the NavMs structure based on these calculations can be seen in the Supplementary Movie 1, with the trajectories superimposed (Fig. 3) on the single representative structure (Fig. 2d).

To confirm the compact nature of the central linker structure indicated by the simulations, a double mutant consisting of A221C at the end of helix S6 and D250C in the coiled-coil region was tested. Its cw-EPR spectrum (Fig. 2a (bottom panel), red line) was comparable with the 1:1 summation (black line) of the spectra of the single mutants A221C and D250C, indicating that labelling at one site does not perturb the structure at the other site. Comparison of the form factor of the double mutant (red line, bottom panel of Fig. 2b) and that of the single mutants (blue line) shows that although the initial shapes of the time evolution curves are almost identical, at later times, the double mutant exhibits quite a different shape to the single mutants, with an additional lower-frequency component and hence the presence of a longer distance (~5 nm). The prediction from the dynamic model (Fig. 2c, black line) gives a similar bimodal distribution of distances to the experimental spectrum (red line) and suggests the linker region is flexible and dynamic, but neither fully extended nor a coiled-coil.

### Comparison of solubilized and membrane-embedded proteins

The cw-EPR spectra of the labels on A221C (at the C-terminal end of helix S6) and A223C (at the beginning of the cytoplasmic domain) were compared (Fig. 2a), for proteins solubilized in detergent micelles (coloured lines) and proteins incorporated into phospholipid membranes (black lines). The aim of this was to investigate whether there was any difference in the mobility, and hence protein structure, in these different environments. These residues were chosen as they are in the parts of the protein that would likely be most susceptible to alterations due to lipid bilayers versus detergent micelles. For each label, the spectra are nearly identical in the two environments, indicating conservation of structure in the detergent-solubilized state.

### Location of the CTD in the crystal structure

**Figure 4:**
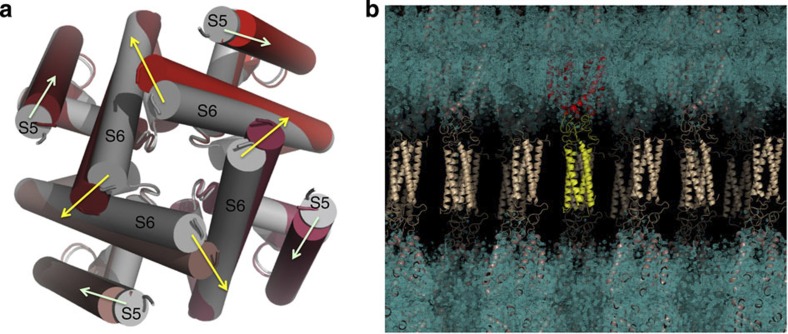
Crystal structure of the NavMs-pore+CTD. (**a**) The tetrameric pore structure of NavMs-pore+CTD is in fully open conformation (depicted in cylinder mode, with each monomer in a different shade of red), overlaid for comparison on the closed channel pore structure of the NavAb orthologue (grey). Yellow arrows show the direction of the movement of the end of S6, and light green arrows the movement of the base of S5, between the closed and open pores. (**b**) Compatibility of the DEER-derived CTD structure with the crystal structure/packing. Electron density map (in blue) overlaid by the structure (in ribbon representation) of the pore domain, with the DEER-defined CTD structure fit into the ‘disordered’ region between ordered tetramers in the crystal lattice. For clarity, one tetramer is depicted with its pore domain in red and its CTD in yellow; the others are in cream.

The 2.9 Å resolution crystal structure of NavMs-pore+CTD (PDB accession code 3ZJZ) (Supplementary Fig. S5) exhibits similar features to the earlier 3.5 Å resolution structure of the NavMs-pore (PDB accession code 4F4L)^9^ in which only half of the CTD was included in the construct (position of truncation indicated by the red arrow in Supplementary Fig. S1). Like the pore-only structure, the present structure exhibits an open pore conformation, but in this case all four monomers are in their fully open state (Fig. 4a). The fully open structure seen here is in a similar conformation to that predicted in our earlier model^9^, which was based on the asymmetric open channel structure. The gating movement (implied by comparison with the closed pore structure in PDB accession code 3RVY) is produced by a change in the Ψ angle of residue Thr84 (numbering according to the NavMs-pore+CTD structure PDBID 3ZJZ, which is equivalent to Thr209 in the full-length sequence), producing an anticlockwise twisting motion of the lower end of the S6 helices and a movement for each of them of ~4.5 Å. The movement of the S6 helices is also accompanied by a slight alteration in the orientation of the amino-acid terminus of the S5 helices, producing a minor displacement (~2.5 A) where they would meet the S4–S5 linker to the voltage sensor domain (Fig. 4a).

In the crystal structure of the NavMs-pore+CTD, there are two crystallographically distinct tetramers. The two tetramers are highly similar, having an RMSD between all Cα atoms of 0.17 Å. Despite symmetry not being enforced, both tetramers have true fourfold symmetry (as was suggested by the DEER measurements), with the distance between the Met97 (equivalent to Met222 in the full-length sequence) Cα atoms at the base of the pore being 21 Å across both diagonals. This is distinctly different from the closed pore in the NavAb structure^7^, where the diagonals are, on average, 13.4 Å. The symmetric tetrameric structure of the NavMs-pore+CTD and wider opening at the bottom of its pore are in agreement with the EPR results for the A221C label at the end of S6. The disorder/flexibility of the CTD observed by EPR (Fig. 3) correlates well with the observed lack of defined density in the electron density map (Fig. 4b), although there are crystallographic indicators of features with some helical order in the domain: inspection of correlation coefficients between the data and the model (provided in BUSTER^18^) reveals that the model alone better accounts for the low-resolution data than the model and solvent scaling together, indicating that a simple continuous electron density does not explain well the regions of the map that do not contain ordered protein atoms. This, taken with the observation that measures of the similarity of symmetry-equivalent reflections (e.g. *R*_merge_, *R*_pim_) showing a characteristic increase (indicative of poorer merging) at ~5.5 Å (the helix repeat distance), suggests that there are helical regions that do not obey the general crystal symmetry and have not been accounted for in the current model. These would be consistent with regular coiled-coil regions attached by a flexible tether, as suggested by Fig. 3. The packing (Fig. 4b) clearly indicates the location of the CTD, and that a disordered linker/coiled-coil structure with the appropriate dimensions would fit in the map, while a fully coiled-coil structure would not. Nevertheless, all attempts at including any of the EPR-derived CTD structures did not produce improved crystallographic R-factors or maps, again suggesting this region to be flexible.

Interestingly, even though this structure contains the full-length CTD, and hence has more unordered residues than the previous NavMs structure^9^, these crystals diffracted to much higher resolution than did the crystals with the truncated CTD.

### Electrophysiological measurements show the CTD is essential

The full-length NavMs channel is a sodium-selective, voltage-gated ion channel with rapid inactivation kinetics^19^. To determine the function of the CTD of NavMs, in addition to full-length channels, expression constructs (Fig. 1a and b arrows) were made that terminated the protein just beyond the end of S6 (Δ223) and at the end of the linker, just before the coiled-coil (Δ239). When transiently expressed in HEK293T cells, in contrast to wild-type channels^19^, all truncated constructs produced little or no sodium currents in whole cell voltage clamp experiments. This result could have arisen either because of the properties of the channel constructs or their inability to traffic to or remain stable in the plasma membrane. As baculovirus infection of insect cells has been successfully used for expression of bacteria sodium channels for electrophysiology characterizations^20^, we then utilized this method to express the NavMs constructs for functional characterization. Conductance of the full-length NavMs channel was enhanced in virally infected Sf9 cells compared with that in transiently transfected HEK293T cells (Supplementary Fig. S6a bottom).

**Figure 5:**
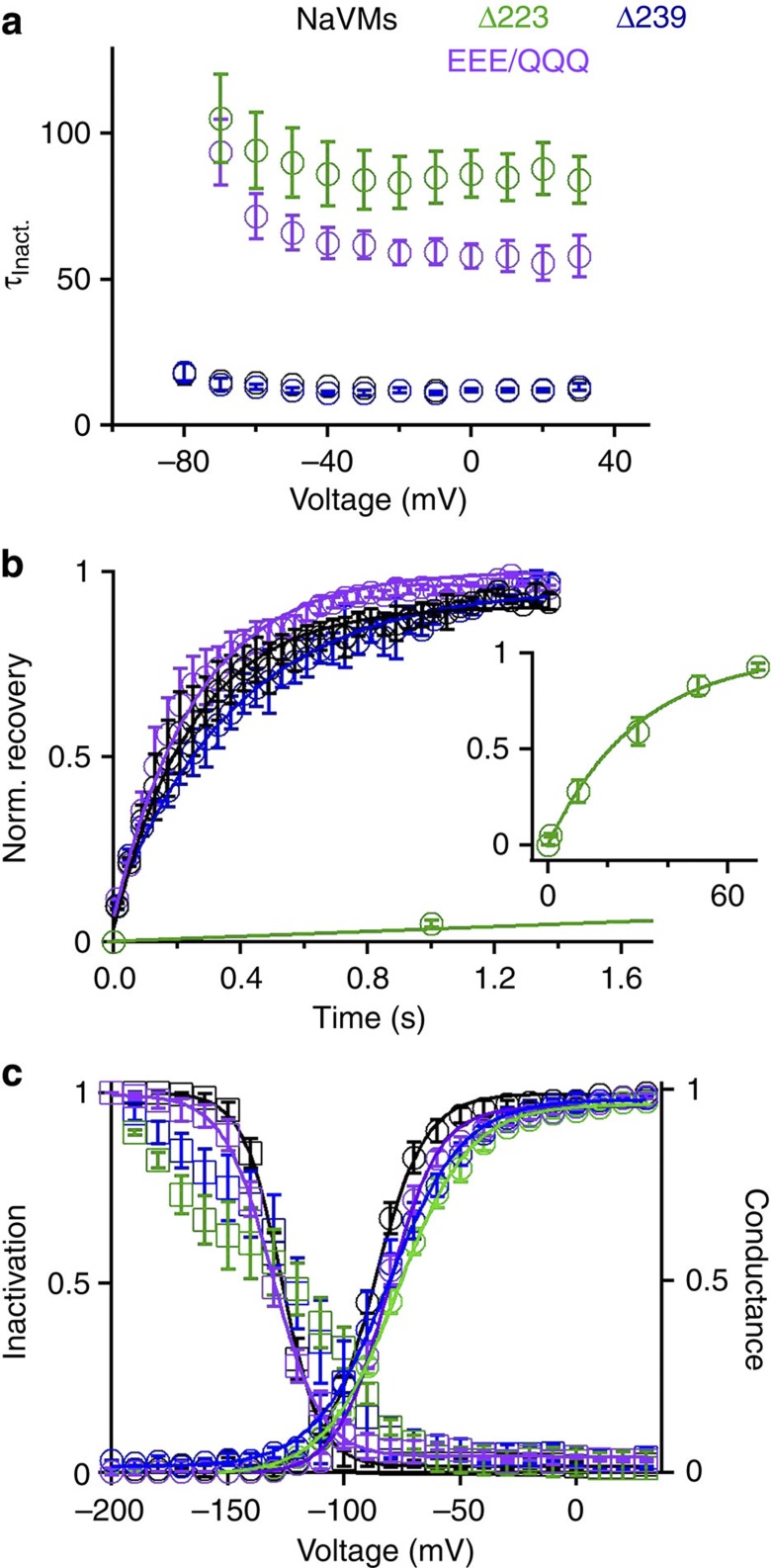
Inactivation properties of wild-type NavMs and truncated and mutant channels in Sf9 cells. (**a**) Time constants of inactivation of the NavMs channels measured from depolarizing voltages from a holding potential of −200 mV. *τ*_inact_ was measured by fitting the decay of current during a 100 or 500 ms depolarization to a single exponential equation. In all panels of this figure, wild-type channel results are shown in black, Δ223 construct in green, Δ239 construct in blue and the EEE to QQQ mutant in purple (error bars=s.e.m.; *n*=4–8 cells). (**b**) Rate of recovery from inactivation. Sodium currents were completely inactivated by a 100 or 500 ms depolarization to −30 mV. The membrane potential was then held at −200 mV for varying time intervals; recovery was assessed by a second depolarization to −30 mV. The proportion of second to the first depolarization is the normalized recovery from inactivation (*n*=4). (Inset) The rate of complete recovery of the Δ223 mutant required a longer time scale (70 s). (**c**) Voltage dependence of activation and inactivation. The steady-state inactivation was assessed by a depolarization to −30 mV after 100 or 500 ms voltage steps. The corresponding conductance was calculated as current divided by voltage (I/V). The resulting conductance–voltage and inactivation–voltage relationships were fit to a sigmoid equation. The voltage dependence of inactivation data of the Δ223 and Δ239 truncations did not converge on a sigmoid relation (error bars=s.e.m.; *n*=4 cells).

When expressed in insect Sf9 cells, the Δ223 and Δ239 truncated channels produced lower-current densities than full-length NavMs (Supplementary Fig. S5b). Inactivation of the Δ223 channel was ~7 times slower than in wild-type NavMs (Fig. 5a, Supplementary Fig. S6c and Supplementary Table S1). Interestingly, the Δ223 inactivation rate is similar to that of the wild-type founding (and functionally best-characterized) member of the voltage-gated sodium channel family, NaChBac^21^. Recovery from inactivation for the Δ223 truncation is ~155 × slower than for wild-type NavMs (Fig. 5b and Supplementary Fig. S6d), requiring a low-pulse frequency (<0.02 Hz) for all voltage protocols performed with this channel. However, the Δ239 construct has the same kinetics of inactivation and recovery as full-length NavMs (Fig. 5a,b and Supplementary Fig. S6c,d). Despite having similar voltage dependence of activation, the voltage dependencies of inactivation of Δ223 and Δ239 were partly uncoupled from the activation process (Fig. 5c and Supplementary Fig. S6e), that is, these truncated channels start to inactivate before opening. The data from the Δ223 and Δ239 constructs indicate that the residues in the linker region, but not those in the coiled-coil, confer inactivation in the NavMs channel, although the coiled-coil restricts the linker so that fast inactivation occurs at the appropriate membrane potential/channel state (after channel opening). Without the coiled-coil, the ‘inactivation linker’ is less coordinated to channel opening as a function of voltage.

The linker region contains a series of negatively charged glutamate residues (E229, E230, E231) (Fig. 1a) that we hypothesized could be involved in rapid inactivation. Mutation of these three residues to glutamines (EEE/QQQ), which preserves the volume and hydrophilic character of glutamate but not the charge, produced a channel that had an ~5 × slower inactivation than wild-type NavMs (Fig. 5a), whereas its voltage dependencies of activation, inactivation and recovery from inactivation were similar (Fig. 5b,c and Supplementary Fig. S6c–e). The current density from the EEE/QQQ mutant was similar to the wild-type NavMs but larger than the Δ223 and Δ239 truncations, both of which lack the coiled-coil domain (Supplementary Fig. S5b). Taken together, these data suggest that the CTD-linker residues E229–E231 are part of a motif that facilitates/enables inactivation, while the coiled-coil motif may instead act to stabilize the channel tetramer.

## Discussion

Previous crystal structures have provided views of the TM regions of tetrameric prokaryotic sodium channels, but not of the CTD, because this domain is dynamically disordered. Synchrotron radiation circular dichroism spectroscopic studies^5^ had determined the secondary structure of the CTD; the EPR, molecular dynamics and new crystal structure described in this work define its supersecondary/quaternary structure and highlight its dynamic nature. Taken together, they demonstrate that the TM domain is connected by a defined but flexible linker region to a C-terminus consisting of a stable four-helix coiled-coil bundle, with one helix contributed by each of the monomers in the tetramer.

Studies of other bacterial sodium channels^6^,^5^ have shown that the CTD can have a role in stabilizing the tetramers, although it is not essential for correct assembly in the membrane. While both the channel and pore-only constructs of NavMs are capable of supporting ion flux without the CTD coiled-coil being present^9^,^22^, electrophysiological studies described in this work show that removal of this region reduces current density (Supplementary Fig. S5a) but not the inactivation rate (Fig. 5a), supporting the notion that the primary role of this motif may be to stabilize the tetramer in the plasma membrane. We also demonstrate that the coiled-coil is essential for coupling inactivation to channel opening. Mutational studies further indicate that the rate of inactivation of NavMs is enhanced by the presence of the flexible linker, and in particular, the three adjacent glutamate residues in this region.

Eukaryotic VGSCs inactivate by both fast (N-type) and slow (C-type) inactivation processes; the fast inactivation has been proposed to involve structural features (in the domain III–domain IV linker) not present in prokaryotic VGSCs. As inactivation gating is also observed in prokaryotic VGSCs, it must therefore arise from a different mechanism. Crystallographic studies^8^,^10^ on prokaryotic orthologues depict structures possessing collapsed selectivity filters, which are of insufficient diameters for the passage of sodium ions, suggesting^8^ that they may represent inactivated states. The large, potentially energetically-costly movements from active to inactive state required by the conformational differences between the crystal structures of open and collapsed selectivity filters have been proposed to be compatible with a slow inactivation process. However, the combination of structural and functional studies in this work, and mutational studies of other prokaryotic orthologues^14^ strongly link the rapid inactivation process in prokaryote VGSCs with changes to the gating (C-terminal) end of the molecule (both the C-terminal end of inner pore helix and the CTD). The model proposed herein is consistent with a low-energy barrier that would facilitate an inactivation/gating of the tetrameric channels in a way that is distinct from eukaryotic VGSCs, and is separate from, but not incompatible with, the C-type inactivation proposed to arise from the collapse of the selectivity filter^8^,^10^ that renders the channel non-conductive.

The sequences of CTDs of all of the prokaryotic sodium channels identified thus far exhibit similar flexible linker/coiled-coil motifs^5^,^6^,^22^. Hence, despite the diversity of ecological niches that their biological sources inhabit, and their different functional roles in their host organisms^3^, these channels have a C-terminal structural feature that is strongly conserved. This would suggest that evolutionary differences in the CTD linker regions (in terms of length and flexibility) and the length of the coiled-coil regions of the different prokaryotic channels may account for the different kinetics of their activation and inactivation processes. Indeed, the rate of inactivation of the NaChBac orthologue (which has a considerably shorter linker region) is 12–19 times slower than that of NavMs (*τ*_inact_=203 ms and 10 ms for NaChBac and NavMs, respectively), and the extent of the inactivation effects due to modifications of the CTD vary for different orthologues^14^.

**Figure 6:**
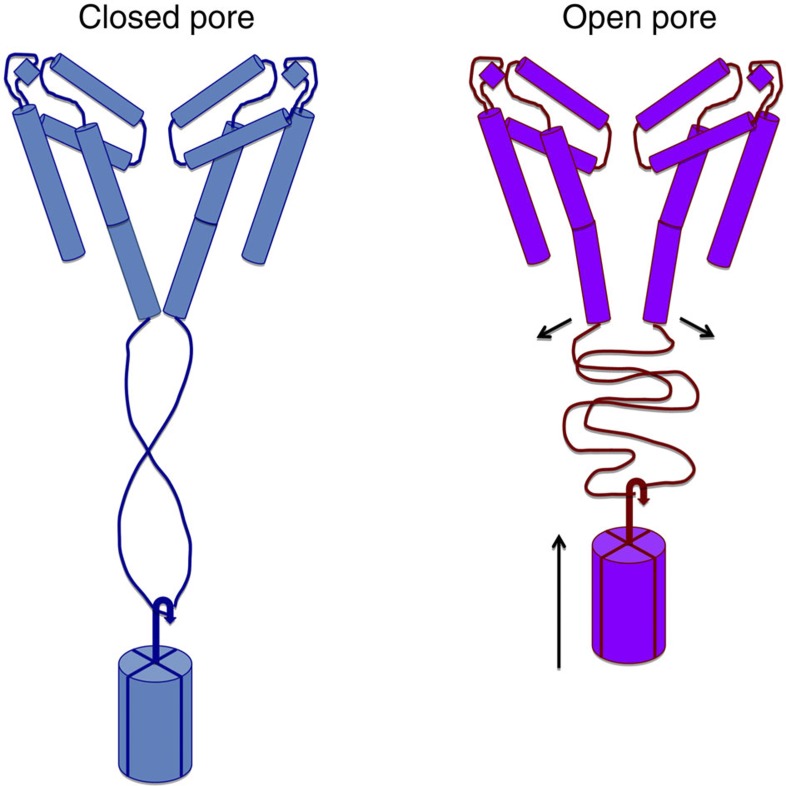
Schematic model for the role of the CTD in gating. The helices in the TM domain are depicted as cylinders, with the break in the middle of the S6 cylinder located at the threonine hinge that produces the difference between open and closed pore structures. Only two monomers of the tetramer are shown for clarity. As an aid to visualisation of extended and relaxed states, the tetrameric coiled-coil at the distal end of the CTD is depicted as a mass on the end of a ‘spring’ formed by the flexible linker region, which can change conformation (without breaking hydrogen bonds) to accommodate the differences in spacing of the ends of the open and closed states.

In summary, structural characterizations of the N-terminal end of the CTD have shown it forms a dynamic but defined structure linking the pore-lining TM helices to the C-terminal coiled-coil bundle. The linker and the coiled-coil regions have different roles in the processes of inactivation channel gating and maintenance of tetramer integrity. This type of structure could enable the opening and closing of the bottom of the pore during gating without disrupting the quaternary structure of the four-helix bundle, a process that would otherwise be energetically very costly. Conformational changes in the linker region, which does not exhibit a regular secondary structure, are unlikely to involve breakage and reformation of a significant number of hydrogen bonds, and so can be accommodated without a major energetic input. The gating can thus be visualized as a type of tethered oscillator enabling the opening of the gate when in the ‘up’ position with the linker in its most flexed conformation, and the closing of the gate in the ‘down’ position with a stretched or extended linker (Fig. 6). Mutations in the EEE motif could destabilize residue–residue interactions with either the S6 gate or the rest of the linker region.

Finally, the CTD structure and mechanism exemplified by these channels may be a paradigm for function, assembly and regulation of voltage-gated ion channels in general. The prokaryotic sodium channels are phylogenetic predecessors of a wide range of tetrameric and dimeric eukaryotic ion channels, all of which apparently retain this motif, including transient receptor potential channels, Kv7 potassium channels, the H^+^ channel Hv1, CatSper calcium channels, PKD2 non-selective ion channels and the 2-pore calcium channels. They all contain C-terminal cytoplasmic motifs that would appear to form similar irregular links to coiled-coil structures. In a number of cases, mutations have been identified in the CTD, which give rise to channelopathies, further emphasizing its functional importance across biological organisms constituting the eukaryotic as well as the prokaryotic domain.

## Methods

### Sequence analysis

Sequence alignment was carried out using ClustalW^23^; the CTD was then manually aligned for optimal positioning of the hydrophobic residues in a and d positions of the heptad repeat. The coiled-coil region was predicted using the programme NCOILS, with a window of 21 residues^24^.

### Expression and purification

The pET15b NavMs-pore+CTD plasmid containing the gene for the wild-type pore+CTD protein (residues G130 to K274) of the NavMs sodium channel from *Magnetococcus marinus* MC-1 was synthesized by Gene Oracle, CA. The cysteine mutants (Fig. 1a) were generated using the QuickChange protocol (Agilent Technologies). Proteins were overexpressed in C41(DE3) cells by the addition of 0.5 mM IPTG at A_600_ _nm_ 0.8 for 3.5 h at 37 °C. After solubilization of the membranes in 1% n-dodecyl-β-D-maltopyranoside (Affymetrix), the proteins were detergent exchanged into 0.52% decanoyl-*N*-hydroxyethylglucamide (HEGA-10) (Affymetrix) and purified using a 1 ml HisTrap HP (GE Healthcare). The His-tags were removed using thrombin and the untagged protein was cleaned on a HisTrap HP. At this stage, the proteins were in 20 mM Tris-HCl, pH 7.5, 300 mM NaCl, 30 mM imidazole and 0.52% HEGA-10.

### Spin labelling and purification of labelled proteins

The cysteines were reduced with 5 mM dithiothreitol (Carbosynth), which was removed by buffer exchange prior to labelling. The proteins were concentrated using a 50 kDa cutoff Amicon Ultracentrifugal filter before size exclusion chromatography (Superdex 200 10/300 GL, GE Healthcare) and elution with buffer A (10 mM Tris-HCl pH 7.5, 100 mM NaCl, 0.52% HEGA-10). They were incubated overnight at 4 °C in the dark, with 3-(2-iodoacetamido)-PROXYL (Aldrich) using a 20-fold molar excess of label-to-protein. Free spin label was removed by size exclusion chromatography in buffer A. They were then exchanged into the same buffer containing D_2_O and 10% glycerol.

Liposome samples were prepared as follows: palmitoyl-2-oleoyl-3-phosphatidylethanolamine and 1-palmitoyl-2-oleoyl-sn-glycero-3-[phosphor-rac-(1-glycerol)] (Avanti Polar Lipids) were mixed in a 3:1 ratio and solubilized in a D_2_O buffer containing 10 mM Tris, pH 7.5, 100 mM NaCl, 0.3% 5-cyclohexyl-1-pentyl-β-D-maltoside (Cymal-5) (Affymetrix), 10% glycerol to produce a lipid concentration of ~7 mM. 50 μl of protein (14 mg ml^−1^) were added to 150 μl of the lipid mixture and incubated for 2 h. Liposomes were formed by adding 20 μl of Calbiosorb beads (Calbiochem).

Samples (concentrations ranging from 150 μM to 200 μM) were transferred into 5 mm NMR tubes (Wilmad) for the DEER experiments and 50 μl capillary tubes for the cw-EPR experiments. The concentrations of spin labels present corresponded to labelling efficiencies in the range of 80–100%.

### EPR spectroscopy

cw-EPR experiments were performed at room temperature on a Bruker EMXplus spectrometer operating at 9.4 GHz equipped with a 4122SHQE resonator. Measurements were performed using 0.2 mW microwave power, 100 kHz modulation frequency, 0.1 mT modulation amplitude and 10 ms conversion time and time constant.

DEER experiments were performed at 50 K on a Bruker ELEXSYS E580 spectrometer operating at 9.6 GHz equipped with an ER-4118-X-MD-5 resonator, Oxford Instruments continuous flow cryostat (CF935) and ITC503 temperature controller. The four-pulse DEER sequence used was *π/*2(*ν*_obs_)-*τ*_*l*_−*π*(*ν*_obs_)−*t′*−*π*(*ν*_pump_)−(*τ*_*l*_+*τ*_*2*_−*t*′)−*π*(*ν*_obs_)−*τ*_2_−echo, where the observer pulse length was 16 ns for *π*/2 and 32 ns for π pulses. The pump pulse length was 12 ns. The long interpulse delay (*τ*_2_) was 3,000 ns for the single mutants and 4,000 ns for the A221C-D250C double mutant. All other parameters were according to Pannier *et al*.^25^ with *τ*_1,0_=400 ns and Δ*τ*_1_=56 ns. Data points were collected in 8 ns time steps. The total measurement time for each sample was in the range of 4–36 h. The spectra (Supplementary Fig. S2) were analysed using the programme DEERAnalysis2011 (ref. 26). The background was corrected by a homology three-dimensional fit and the distance distributions evaluated by either Tikhonov regularization or two Gaussian distance distributions or two Rice distance distributions. The damped oscillation reached a plateau by 0.5 μs.

### Modelling and DEER simulations

The initial homology model of the NavMs-pore TM domain was built based on the symmetric tetramer model derived from monomer A of the NavMs open pore crystal structure (PDBID=4F4L)^9^ using MODELLER software^27^. The structure of the CTD was first modelled assuming that the entire CTD was a coiled-coil, using residues 9–22 from PDBID 2CCE^28^ as a template (Supplementary Fig. S4a). The register of the coiled-coil was determined by aligning the NavMs sequence with that of NaK-NavSulP (Fig. 1a).

In the first dynamics model, 200 ns coarse grained molecular dynamics simulations were performed using the MARTINI force field^29^,^30^ at 323 K in a pure DPPC bilayer using the Berendsen weak-coupling algorithm to couple temperature and pressure. The secondary structure of the connecting regions between the pore and the coiled-coil domain was specified as being random coil. One-thousand CG protein structures from this simulation were converted to all-atom representations using MODELLER, and the DEER distributions for each structure were predicted using MMM^17^, before being summed to create a single distribution. To create a final improved model, the conversion and refinement to the all-atoms model was repeated, with flexible regions at the tips of the coiled-coil relaxed to enable disordering. A schematic diagram of the refinement process is shown in Supplementary Fig. S3.

### Crystallization and crystal structure analysis

For crystallization, the untagged wild-type protein purified after the second HisTrap HP was concentrated before loading onto the size exclusion column in buffer A, concentrated to 14 mg ml^−1^ and used directly or frozen at −80 °C. Crystals were grown at 4 °C using the vapour diffusion method. The protein was mixed in a 2:1 ratio with reservoir solution containing 0.1 M trisodium citrate, 0.1 M Tris-HCl, pH8, 34% v/v PEG400 for 150 nl sitting drops. Data sets were collected at the Diamond Light Source. The final data set was scaled and integrated with iMosflm^31^ and Aimless^32^ and the structure was determined with Phaser^33^ using monomer A from the NavMs-pore structure (PDB accession code 4F4L). Refinement was carried out with Buster^18^ and Phenix^34^. The data collection, processing and refinement statistics are listed in Table 1.

### Electrophysiology

Insect cell virus containing the recombinant NavMs channel gene was generated using the Bac-to-Bac TOPO Expression System (Invitrogen). The *NavMs* gene was cloned into the pFastBac1 donor plasmid and transformed into DH10Bac *Escherichia coli* to produce NavMs Bacmid DNA. *Spodoptera frugiperda* ovarian cells (Sf9) were then transfected using Cellfectin II (Invitrogen) with the recombinant NavMs Bacmid to generate the first generation of NavMs–baculovirus. After two rounds of viral propagation, high-titer (1 × 10^7^to 1 × 10^8^ pfu ml^−1^) recombinant baculovirus stocks were used for infection. Infected cells were whole cell voltage clamped 36–48 h after infection. In many cases, currents from the Sf9 cells were too large to accurately voltage clamp and occasionally saturated the amplifier (>20 nA). Currents larger than 9 nA were disqualified due to excessive voltage error. Cells were patched with low-resistance pipettes (≤2.5 mΩ) in order to keep the voltage error below 4 mV. Extracellular solutions contained (in mM), NaCl,150; CaCl_2_,1.8; MgCl_2_, 1; HEPES, 10; pH 7.4. The intracellular (pipette) solution contained (in mM) the following: CsF,105; NaCl, 30; HEPES, 10; BAPTA-Cs, 5; MgCl_2_, 2; pH 7.3. CaCl_2_ was added to achieve 100 nM free Ca^2+^. Data were analysed by Igor Pro 6.00 (Wavemetrics, Lake Oswego, OR). Residual leak (>−100 pA) and capacitance were subtracted using a standard P/-4 protocol. Current–voltage relationships were fit with (*V*−*V*_rev_)/{1+exp[(*V*−*V*_1/2_)/*k*]} where *V*_rev_ is the extrapolated reversal potential. The equation for the exponential fits used in Fig. 5a,b was: *f*(*x*)=*B*+*A *exp[(1/*τ*)*x*], where *τ* is the half-time constant current decay or recovery rate and *x* is time. Voltage protocols testing the Δ223 channel were repeated at 0.014 Hz to allow for recovery from inactivation, whereas those used for all other channels were repeated at 0.1 Hz.

## Author contributions

C.B. performed the molecular biology, purified and labelled the proteins, produced and screened the crystals. C.E.N. and C.B. undertook the structure solution and refinement. C.B. and C.W.M.K. collected and analysed the EPR data, and B.A.H. built and refined the EPR structure. C.B., C.E.N., C.W.M.K. and B.A.W. analysed the structures. P.G.D. made the constructs and conducted the electrophysiological studies, and together with D.E.C. analysed the electrophysiology results. C.B. and B.A.W. wrote the initial draft of the manuscript, which was discussed, modified and approved by all authors. C.B., C.W.M.K. and B.A.W. initiated the project. B.A.W. and C.W.M.K. supervised the project and are co-corresponding authors.

## Additional information

**Accession codes**: Atomic coordinates and structure factors have been deposited in the Protein Data Bank under accession code 3ZJZ.

**How to cite this article:** Bagnéris, C. *et al.* Role of the C-terminal domain in the structure and function of tetrameric sodium channels. *Nat. Commun.* 4:2465 doi: 10.1038/ncomms3465 (2013).

## Supplementary Material

Supplementary Figures and Table Supplementary Figures S1-S6 and Supplementary Table S1

Supplementary Movie 1 Dynamics of the NavMspore+CTD. 200 ns coarse grained molecular dynamics simulation demonstrating the bending and flexibility of the connection region between the pore and coiled-coil in the CTD. The pore (red) and CTD (yellow) are rendered as backbone ribbons.

## Figures and Tables

**Table 1:** Data collection and refinement statistics for NavMs-pore+CTD.

*Data collection*	
Space group	*C222*_*1*_
Cell dimensions	
*a*, *b*, *c* (Å)	80.15, 334.38, 80.21
*α, β, γ* (°)	90.0, 90.0, 90.0
Resolution (Å)	50.0-2.9 (3.10–2.9)*
*R*_sym_ or *R*_merge_	19.6 (68.1)
*I*/*σI*	11.9 (4.1)
Completeness (%)	99.7 (98.6)
Redundancy	13.4 (13.9)
	
*Refinement*	
Resolution (Å)	58–2.90
No. reflections	23860
*R*_work/_ *R*_free_	25.7/28.7
No. atoms	
Protein	2880
Ligand/ion	394
Water	154
B-factors	
Protein	51.36
Ligand/ion	80.75
Water	38.37
R.m.s. deviations	
Bond lengths (Å)	0.010
Bond angles (°)	1.10

^*^Highest resolution shell is shown in parenthesis.
