# Supplementary material for: Role of the C-terminal domain in the structure and function of tetrameric sodium channels
Source: Nat Commun. 2013 Sep 19;4:2465. doi: 10.1038/ncomms3465 (PMC3791462; doi:10.1038/ncomms3465)
Supplement: Supplementary Figures and Table — Supplementary Figures S1-S6 and Supplementary Table S1 [file ncomms3465-s1.pdf]

Supplementary Figures:

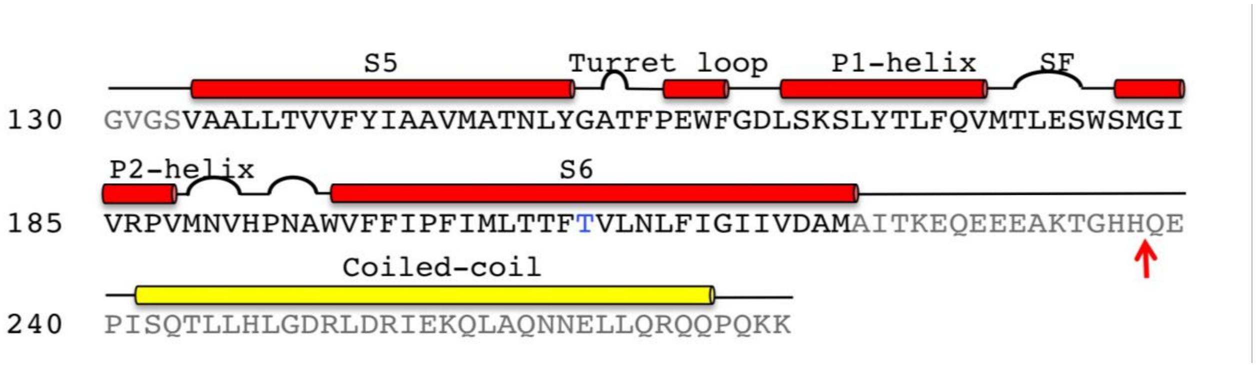

Supplementary Figure S1. The NavMs-pore+CTD Sequence.
The sequence of the construct, with the helical regions in the transmembrane pore domain indicated by the red bars and the coiled-coil helix in the CTD indicated by the yellow bar. The arrow shows the terminus of the "half-length" CTD. The residues in grey are not visible in the electron density map.

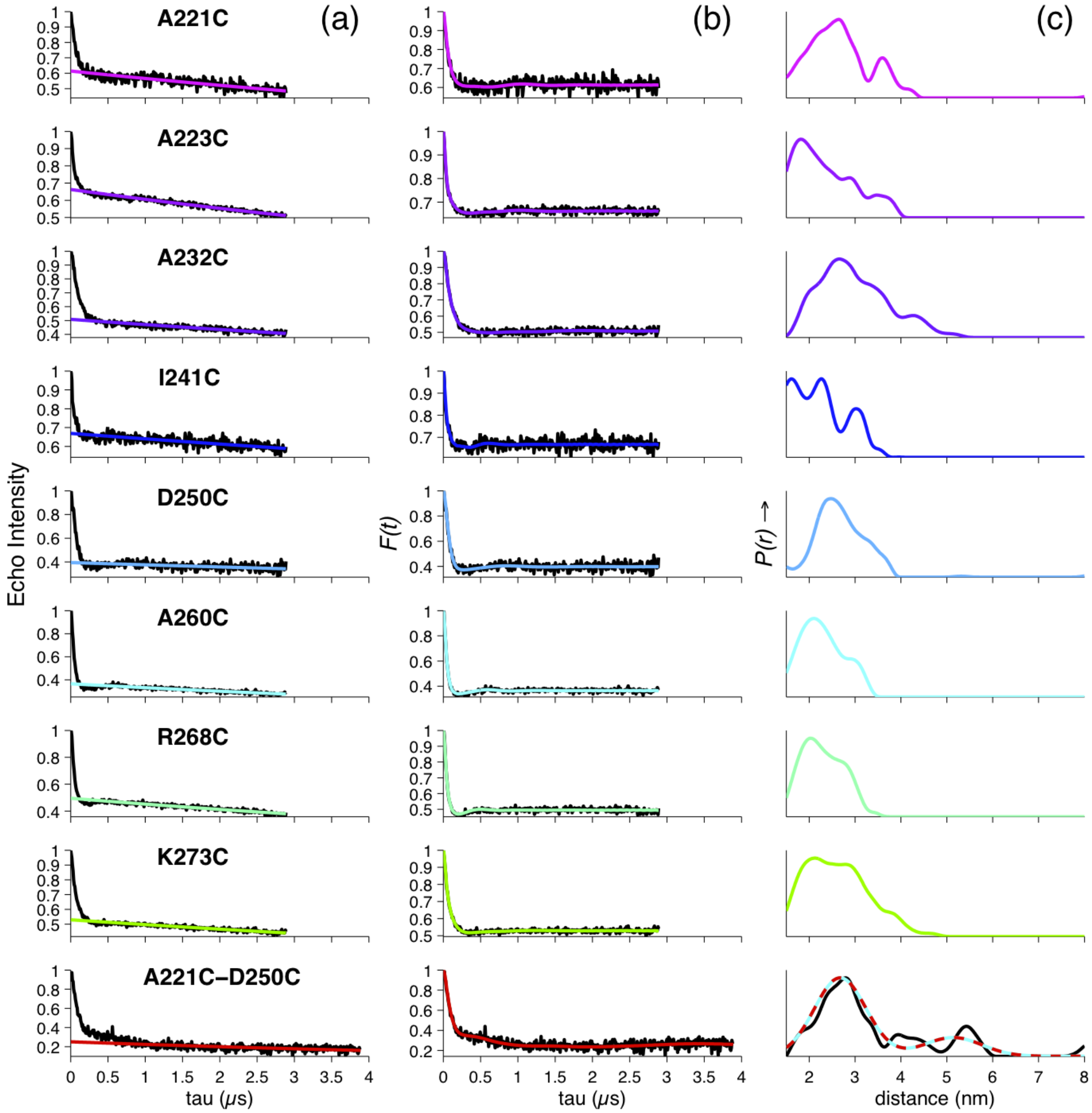

Supplementary Figure S2. DEER Measurements of the Distances Between Four Nitroxide Spin Labels in the NavMs Mutants.

a. Raw data (black lines) and background decay (coloured lines). The raw data are divided by the decay function to obtain the plots in b.

b. Background corrected dipolar evolution data (black lines). Coloured lines are fits to the data obtained by Tikhonov regularization giving the distance distributions in c.

c. Distance distributions obtained by Tikhonov regularization (coloured lines). The A221C-D250C double mutant (bottom panel) has additionally been fitted with two Gaussian distributions (cyan line) and two Rice distributions (red line). These distance distributions are identical and are similar to that obtained by Tikhonov regularization (black line). All plots are normalized by amplitude.

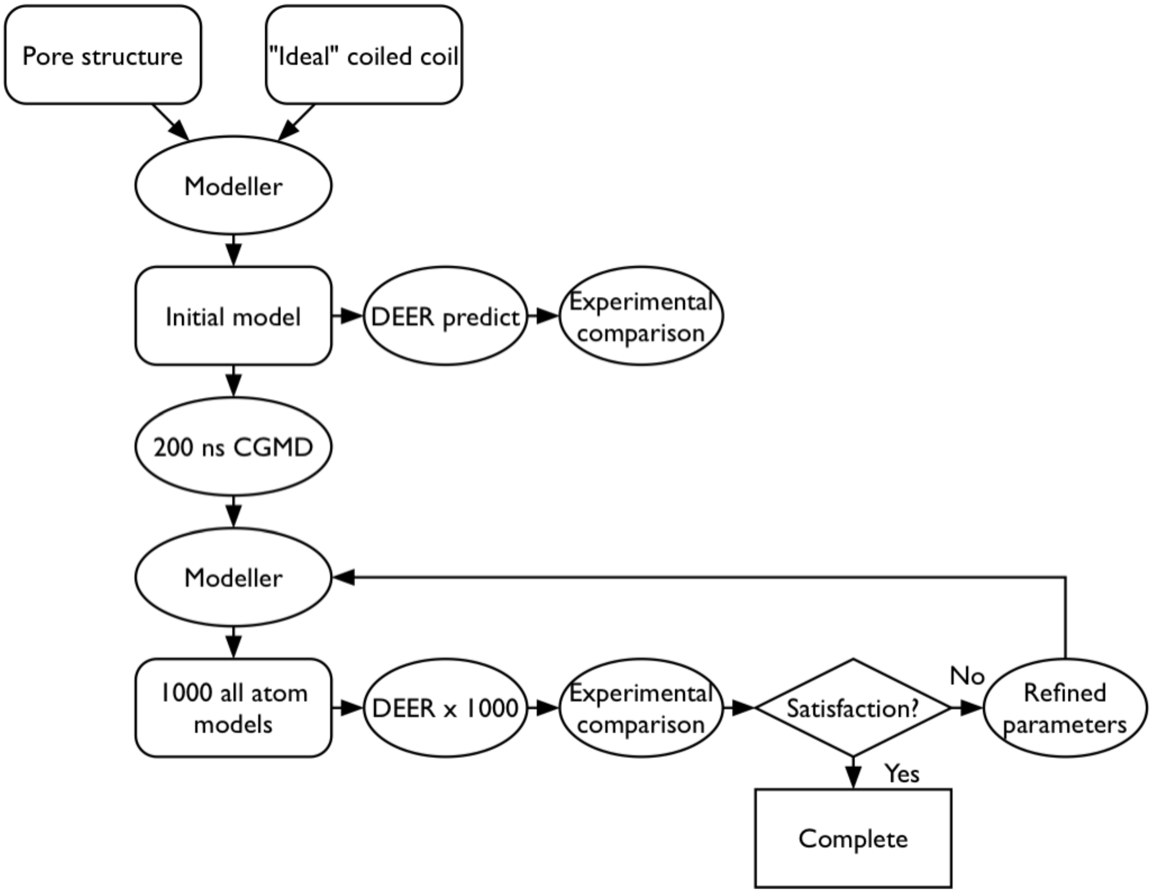

Supplementary Figure S3. Schematic of Workflow used in the DEER Modelling and Refinement.

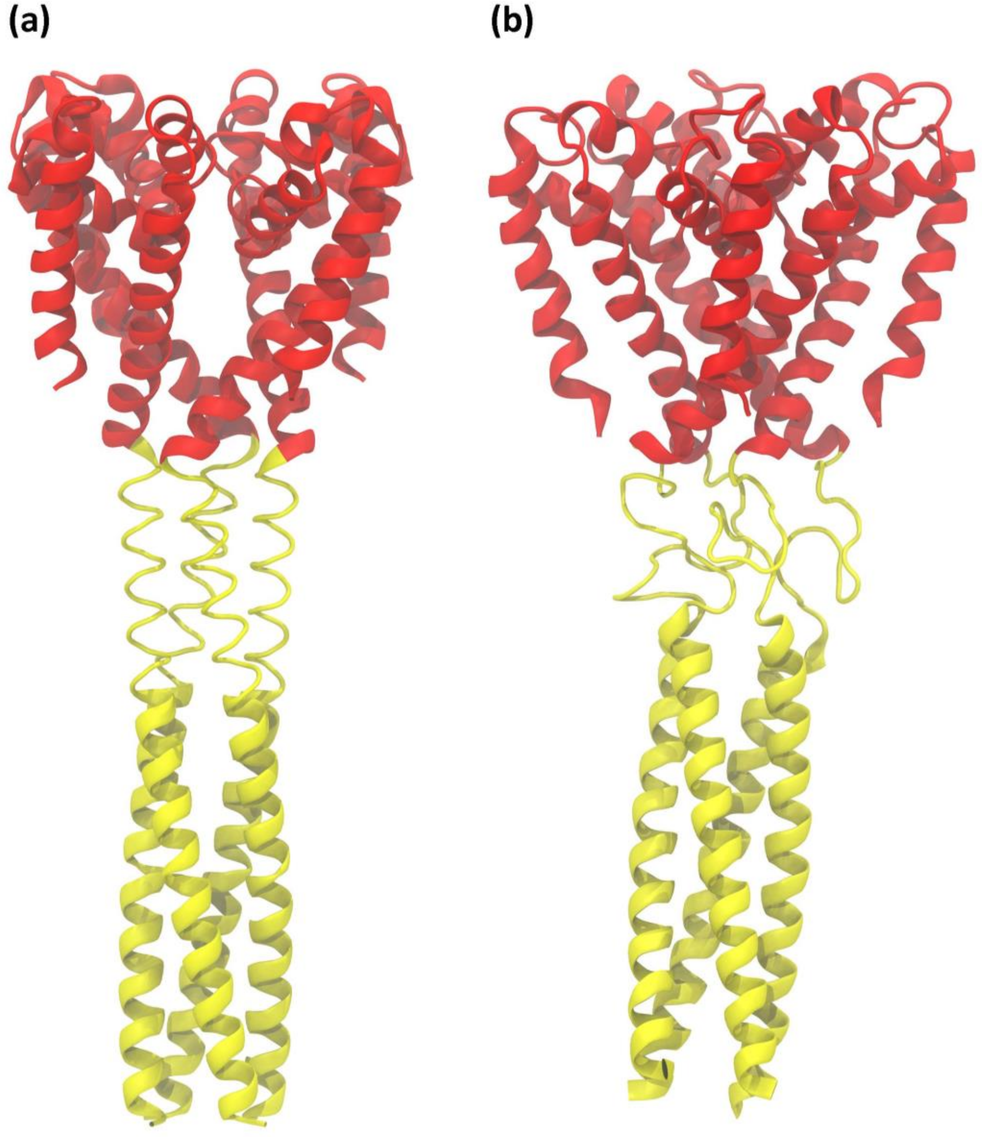

Supplementary Figure S4. Models Used in the First Two Steps of Optimisation of the DEER Data Fitting.
a. Initial static model constructed by connecting a completely coiled-coil region (based on the structure of 9 to 22 from pdb code 2CCE *(26)*) to the end of helix S6 in the symmetric tetramer NavMs structure *(9)*.
b. One frame of the first dynamic model created by applying 200 ns MARTINI CG simulations in a dipalmitoylphosphatidylcholine membrane. The residues in the flexible connecting region were allowed to relax from their initial helical conformations. Over the 200 ns simulation the TM and coiled-coil regions maintained their folds, whilst the connecting region quickly relaxes to an unstructured state.

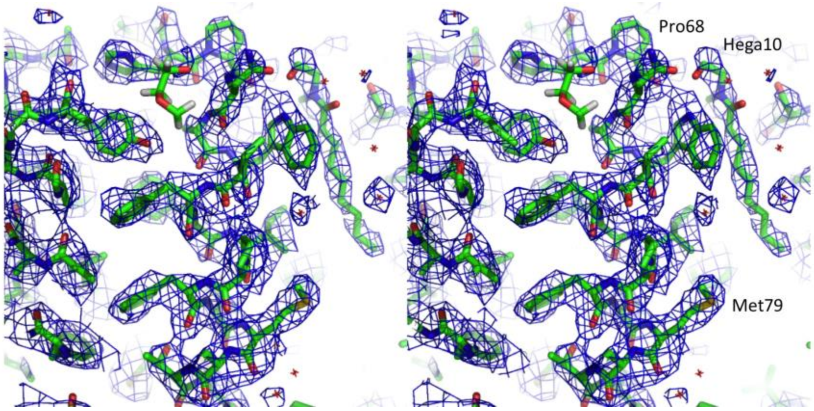

Supplementary Figure S5. Example of Electron Density Map.
Stereo representation of the 2.9 Å resolution electron density 2Fo-Fc map contoured at 1.5 σ, highlighting residues at the top of the S6 domain (molecule B) and one Hega10 detergent molecule in stick representation.

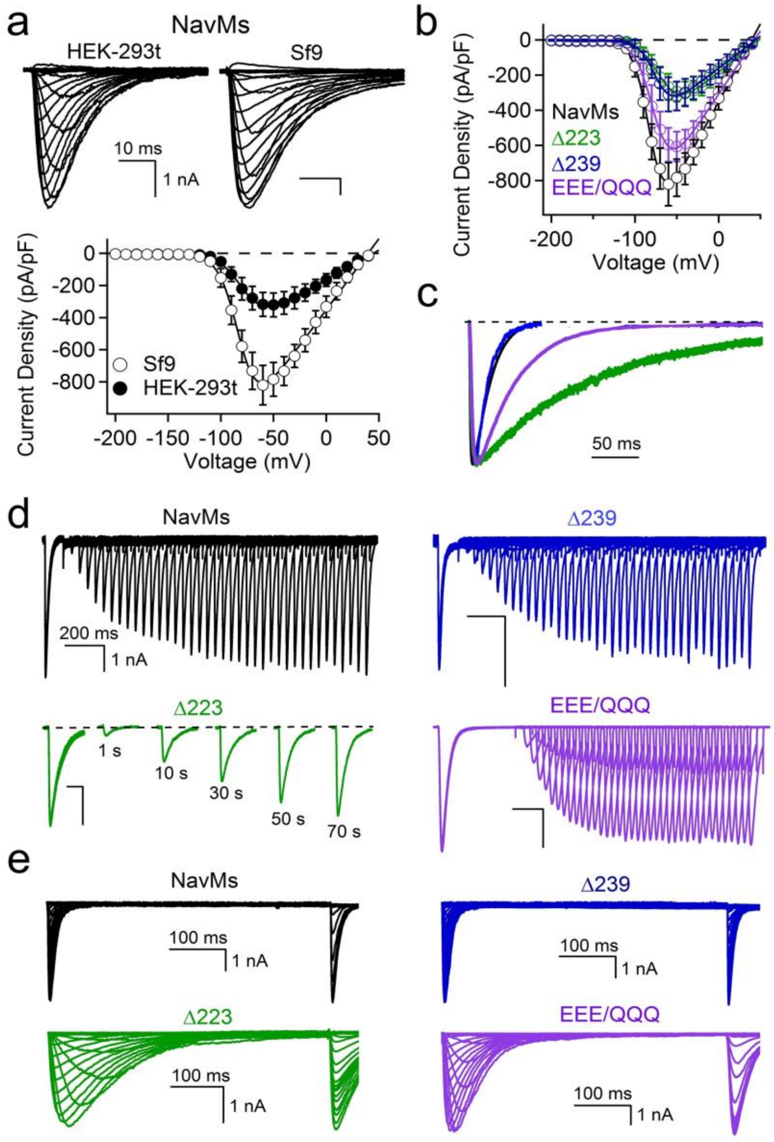

Supplementary Figure S6. Electrophysiological characterisation of NavMs channels.

a. NavMs whole cell current densities recorded from HEK-293T and SF9 cells. (top) Example sodium current traces activated by increasing 100 ms depolarizations from a -200 mV holding potential. (bottom) Resulting current densities as a function of voltage from wildtype NavMs expressed in HEK293T and SF9 cells (n = 6-8 cells; Error bars = SEM).

b. Sodium current densities recorded from NavMs channel constructs expressed in SF9 cells. (n = 5-7, Error bars = SEM). In all panels of this figure, wildtype channel results are shown in black, Δ223 construct in green, Δ239 construct in blue, and the EEE to QQQ mutant in purple.

c. Example current traces comparing the inactivation rates of the NavMs channel constructs. Normalized current amplitudes were activated by a -30 mV depolarization.

d. Current records testing the recovery from inactivation by the NavMs channel constructs. The inactivating pulse is on the left side and each subsequent test pulse is found to the right side of the current records. Due to the long recovery time, the Δ223 current record was abbreviated for clarity.

e. Example current records of the voltage dependence of inactivation and activation. For each channel, the left side currents are activated by 500 ms depolarizing voltage steps from -200 to 40 mV. The right side currents were elicited by the test pulse of -30 mV.

Supplementary Table:

Channel	Activation $V_{1/2}$ (mV)	Inactivation $V_{1/2}$ (mV)	τ_{inact} (ms)	$\tau_{recovery}$ (ms or s)
**NaVMs HEK-293T**	-85 ± 3	-120 ± 3	10 ± 1	209 ms ± 4
**NaVMs SF9**	-88 ± 2	-125 ± 2	13 ± 2	190 ms ± 6
**Δ223**	-77 ± 3	-	88 ± 7	29.4 s ± 3
**Δ239**	-82 ± 2	-	12 ± 2	205 ms ± 11
**EEE/QQQ**	-80 ± 2	-131 ± 3	66 ± 3	179 ms ± 12

Supplementary Table S1. NavMs channel properties measured using the whole cell patch clamp.
Voltage dependence of activation (Activation V1/2); voltage dependence of inactivation (Inactivation $V_{1/2}$); Time constant of inactivation (τ_{inact}) measured at -30mV. Time constant of recovery from inactivation (τ_{inact}). ± values represent SEM of 6-8 replicates.
